# A Randomized, Double-Blind, Placebo-Controlled, Multicentered Study to Evaluate the Efficacy and Safety of MEI005 in Reducing Submental Fat in Chinese Adults

**DOI:** 10.1093/asj/sjaf031

**Published:** 2025-03-04

**Authors:** Wenyun Ting, Junxian Wen, Zhijin Li, Jiaming Sun, Jiaping Zhang, Maoguo Shu, Hongwei Liu, Li He, Bo Yu, Nan Jiang, Chunyu Xue, Qian Tan, Xuewen Xu, Sufan Wu, Dan Jian, Hongyi Zhao, Lei Wang, Nanze Yu, Xiaojun Wang

## Abstract

**Background:**

Submental fat (SMF) is a common aesthetic concern traditionally treated with liposuction. Nonsurgical alternatives, like injectable deoxycholic acid, are gaining popularity. However, no related products have been clinically approved in China.

**Objectives:**

This study evaluated the efficacy and safety of MEI005, a deoxycholic acid–based injectable formulation, in reducing SMF in Chinese adults.

**Methods:**

This multicenter, randomized, double-blind, placebo-controlled Phase III trial included 325 Chinese adults with moderate-to-severe SMF. Participants received MEI005 or a placebo injection every 28 days for up to 6 sessions. Primary endpoints were ≥2-grade improvements in Clinician and Patient-Reported Submental Fat Rating Scales (CR-SMFRS and PR-SMFRS). Secondary endpoints included SMF volume reductions measured by MRI; patient-reported outcomes on the Patient-Reported Submental Fat Impact Scale (PR-SMFIS) and Subject Self-Rating Scale (SSRS); and SMF thickness reductions measured by caliper. Adverse events (AEs), self-reports, clinical examinations, and checks for skin laxity were monitored.

**Results:**

At 12 weeks posttreatment, 18.9% of MEI005 recipients demonstrated simultaneously ≥2-grade CR-SMFRS and PR-SMFRS improvement vs 1.8% for placebo (*P* < .001). Additionally, 68.9% of MEI005 group participants achieved simultaneously ≥1-grade improvement in CR-SMFRS and PR-SMFRS, compared with 21.6% of placebo (*P* < .001). MRI revealed ≥10% SMF volume reduction in 50% of MEI005 patients vs 15.2% of placebo (*P* < .001). Scores on the PR-SMFIS and SSRSs showed greater psychological improvement and satisfaction among MEI005 recipients. Vernier caliper measurements showed a 21.42% thickness reduction vs 6.32% (*P* < .001). AEs were mostly mild to moderate.

**Conclusions:**

MEI005 effectively reduces SMF, offering a safe, minimally invasive option for Chinese adults.

**Level of Evidence: 2:**

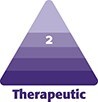

Submental fat (SMF), commonly known as a double chin, refers to the accumulation of fat in the preplatysmal compartment of the neck. This fat is often viewed as undesirable in appearance, as it can obscure jawline definition and negatively impact patients’ confidence and self-esteem.^1^ Previously, open surgical procedures, such as submental lipectomy, platysmaplasty, and direct excision of excess tissue, were performed to remove excess fat and reshape the neck.^2-4^ Over time, liposuction became a frequently used surgical method to remove excess SMF and improve the contour of the neck and chin. However, surgery involves significant risks, including skin contour irregularities, bruising, local infections, nerve injury, and, in some cases, prolonged recovery times.^5-7^ Consequently, less invasive, nonsurgical skin-tightening procedures—such as laser therapy, radiofrequency, high-intensity focused ultrasound, and fat-dissolving injections—have gained popularity in neck rejuvenation.^8-10^

ATX101 (Kybella/Belkyra, Allergan, Irvine, CA), a deoxycholic acid injection, is the only injectable fat-dissolving product approved by the FDA and European Medicines Agency (EMA). Since its approval, it has been marketed in various countries, including Canada, Australia, Singapore, and South Korea. Four Phase III clinical trials have demonstrated the clinical efficacy and safety of ATX101 in reducing SMF.^11-14^ However, there are currently no officially approved fat-dissolving injections in mainland China. The market there is filled with numerous unregulated products lacking systematic clinical research data, posing significant safety risks.

A new injectable lipolytic drug, MEI005 (2017L04493), was developed in China to improve the appearance of moderate-to-severe convexity or fullness associated with SMF in adults. Its active ingredient is also deoxycholic acid. In this randomized, double-blind, placebo-controlled, multicenter Phase III clinical trial (CTR20212274), we aimed to evaluate the efficacy and safety of MEI005 in reducing SMF in Chinese adults.

## METHODS

### Trial Design

This multicenter, randomized, double-blind, parallel group, placebo-controlled clinical trial was conducted at 15 sites in China from January 2022 to May 2023, in compliance with regulations set by the Chinese FDA. All potential patients voluntarily signed an informed consent form (ICF) approved by the ethics committee. After signing the ICF, patients entered a screening period (Day −14 to Day −1), during which eligibility was assessed based on inclusion and exclusion criteria. Eligible participants were then randomized in a 2:1 ratio to receive either MEI005 (10 mg/mL, Nanjing Minova Pharmaceutical Co., Ltd) or a placebo composed of inactive ingredients, including phosphate, sodium chloride, sodium hydroxide, and hydrochloric acid.

To ensure safety and comfort, a compound lidocaine cream was applied to the submental area for local anesthesia before injection. A 30 G, 13 mm needle was used to inject 0.2 mL of the trial drug into the preplatysmal fat at each marked site (Supplemental Figure 1), maintaining a 1.0 cm interval between injection points (2 mg/cm^2^). Each patient received a maximum of 10 mL of injection fluid per treatment session. After the injection, patients were monitored in the hospital for at least 30 min for safety assessment.

**Figure 1.**
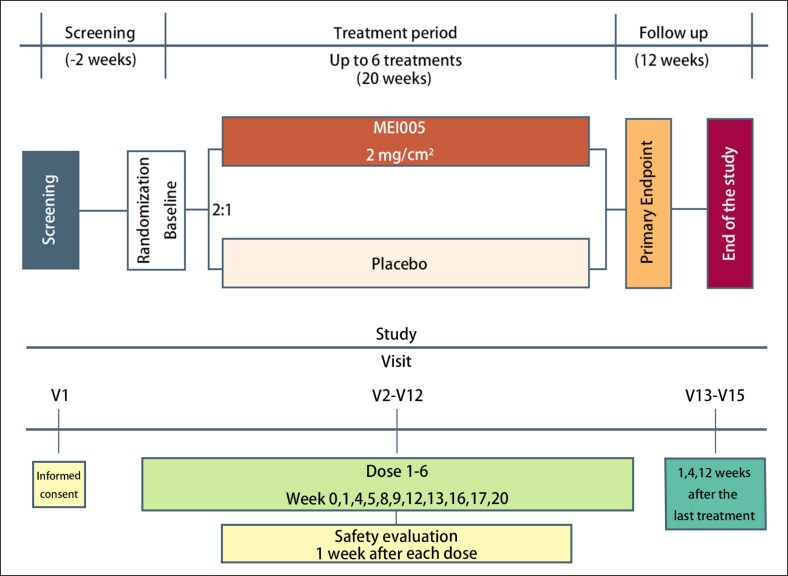
Diagram outlining the study design.

Treatments were administered at intervals of 28 days (±5 days). If participants experienced symptoms, such as redness, swelling, pain, menstrual period, local adverse reactions, or general discomfort, treatment could be delayed for up to 2 weeks at the investigator's discretion. The maximum number of treatments was limited to 6, based on considerations of patient safety and clinical efficacy. The diagram outlining the study design is shown in Figure 1.

### Patients

Eligibility criteria included adults aged 18 to 65, of any gender, with moderate-to-severe SMF accumulation graded as 2 or 3 on the validated Clinician-Reported SMF Rating Scale (Supplemental Figure 2). During screening, participants were required to have a BMI between 17 and 40 kg/m^2^, with stable body weight over the preceding 6 months and fluctuations not exceeding ±10%. Body weight was measured at each follow-up visit during the treatment period, and participants with weight fluctuations ≥10% were subjected to sensitivity analysis during statistical evaluation. All were required to have no plans for pregnancy during the study period.

Exclusion criteria encompassed a history of surgery, liposuction, or injections of lipolytic agents in the submental area; individuals who had received dermal fillers or botulinum toxin injections in the neck within the previous 3 months or had plans for cosmetic procedures in the submental area; patients with submental enlargement because of causes other than fat accumulation—such as thyroid or lymph node enlargement, Madelung's disease, or prominent sternocleidomastoid muscles; patients with visible scars, active infections, cancerous or precancerous lesions, unhealed wounds, retrognathia, or other relevant conditions. Participants with a Grade 4 on the Submental Skin Laxity Grade (SMSLG) scale or anatomical features that might lead to suboptimal cosmetic outcomes, as well as individuals prone to hypertrophic scarring or keloids; participants with acute or chronic illnesses, bleeding disorders or abnormal coagulation profiles, and infectious diseases; participants who were plans to undergo procedures or take medications during the study that could lead to significant weight changes (≥10%; eg, systemic corticosteroids, weight-loss medications, or bariatric surgery); participants with a history of substance abuse, significant blood loss (≥200 mL), or blood donation within the past 3 months; participants with known allergies to deoxycholic acid, any study drug components, or lidocaine; participants who had taken part in any other clinical trials involving investigational drugs or devices within the past 3 months were also excluded. Investigators would exclude participants if any condition was deemed to compromise safety or interfere with the integrity of study results.

### Trial Outcomes

The primary efficacy endpoints were defined as: (1) a simultaneous improvement of at least 2 grades on both the Clinician-Reported Submental Fat Rating Scale (CR-SMFRS) and the Patient-Reported Submental Fat Rating Scale (PR-SMFRS) 12 weeks after the final treatment, and (2) a simultaneous improvement of at least 1 grade on both scales.

The secondary efficacy endpoints included: (1) the proportion of patients showing at least a 10% reduction in submental volume from baseline, confirmed by magnetic resonance imaging (MRI) 12 weeks postfinal treatment. All MRI measurements were performed at a single MRI center in China. The SMF region, located below the mandible to the hyoid bone and bordered by the digastric or stylohyoid muscles, was measured by outlining the region of interest (ROI) on sagittal MRI slices (Supplemental Figure 3). Ten consecutive 1 mm slices were traced, covering a total thickness of 10 mm. The MR MultiParametric Analysis software (V1.2.1, Siemens Healthcare, Erlangen, Germany) automatically excluded nonfat tissues, and the total SMF volume was calculated by summing the ROI areas across the 10 slices and multiplying by the slice interval. The results were recorded in milliliters with a precision of 0.1 mL, and data were saved in both screenshot and DICOM formats; (2) assessment of the psychological impact of SMF through changes in the Total Scale Score on the Patient-Reported Submental Fat Impact Scale (PR-SMFIS); and (3) reduction in SMF thickness from baseline measured by vernier calipers. Additional efficacy endpoints included: (1) differences in satisfaction scores, measured by the Subject Self-Rating Scale (SSRS); (2) differences in the proportion of participants in the experimental and control groups achieving a 1- or 2-grade improvement on the CR-SMFRS 12 weeks after the final treatment; (3) differences in the proportion of participants in the experimental and control groups achieving a 1- or 2-grade improvement on the PR-SMFRS at 12 weeks postfinal treatment; and (4) differences in the proportion of participants in the experimental and control groups reporting effective responses to the Subject Global Questions (SGQs) at 12 weeks postfinal treatment. All secondary endpoints were assessed 12 weeks after the final treatment, and all scales used in this study are summarized in Table 1.

**Table 1.** Scales Used in This Study

Scales	Description
CR-SMFRS	Submental convexity was evaluated by the clinician on a 5-point ordinal scale (Supplemental Figure 1): 0, absent; 1, mild; 2, moderate; 3, severe; 4, extreme
PR-SMFRS	SMF evaluated by patient on a 5-point ordinal scale: 0, no chin fat at all; 1, a slight amount of chin fat; 2, a moderate amount of chin fat; 3, a large amount of chin fat; 4, a very large amount of chin fat
PR-SMFIS	The psychological impact of SMF on self-perception of 6 emotional and visual characteristics related to the appearance of submental fullness, assessed with the following items: How happy are you with the appearance of your chin fat? How bothered are you by the appearance of your chin fat? How self-conscious are you about the appearance of your chin fat? How embarrassed are you about the appearance of your chin fat? How much older do you look because of your chin fat? How much overweight do you look because of your chin fat? Each item rated on an 11-point numeric scale (0-10) and scores for the 6 items were combined to generate a PR-SMFIS total scale score. Lower scores indicate improvement or reduced negative impact of these items
SSRS	Overall satisfaction with the appearance of the face and chin was evaluated by the patient on a 7-point scale: 0, extremely dissatisfied; 1, dissatisfied; 2, slightly dissatisfied; 3, neither satisfied nor dissatisfied; 4, slightly satisfied; 5, satisfied; 6, extremely satisfied A responder was a patient whose response was 4, 5, or 6
SGQ	Patients evaluated the fat under the chin (SGQ1), the definition between the chin and neck (SGQ2) compared with before treatment, and their satisfaction with the treatment (SGQ3) Options for SGQ1 and SGQ2: a great deal worse; moderately worse; a little worse; about the same; a little better; moderately better; a great deal better Options for SGQ3: extremely dissatisfied; moderately dissatisfied; a little dissatisfied; neither dissatisfied nor satisfied; a little satisfied; moderately satisfied; extremely satisfied A responder was 1 of the 2 highest positive categories
SMSLG	A responder was defined as a patient whose response fell into 1 of the 2 highest positive categories. Skin laxity was assessed by a clinician based on skin wrinkling, adherence to underlying neck structures (bone and muscle), and redundancy (horizontal and vertical folds), using a 4-point scale: 1, none; 2, mild; 3, moderate; 4, severe

CR-SMFRS, Clinician-Reported Submental Fat Rating Scale; PR-SMFIS, Patient-Reported Submental Fat Impact Scale; PR-SMFRS, Patient-Reported Submental Fat Rating Scale; SGQ, Subject Global Questions; SMSLG, Submental Skin Laxity Grade; SSRS, Subject Self-Rating Scale.

### Safety Evaluation

Safety evaluations were conducted through outpatient follow-ups and phone calls to monitor adverse reactions 1 week after each injection. At each follow-up, vital signs and body weight were recorded. Laboratory tests—including complete blood counts, general chemistry, coagulation tests, urinalysis, and thyroid-stimulating hormone levels—were conducted at the screening visit, with additional blood counts and general chemistry tests at Weeks 4, 12, and 20. A blood pregnancy test was also performed at the screening visit and at these time points.

Adverse events (AEs) were assessed through both self-reports and physician examinations. Special attention was given to treatment-site reactions, such as edema, swelling, hematoma, bruising, erythema, pigmentation, induration, numbness, pain, paresthesia, pruritus, dysphagia, voice disturbances, nerve injury, and allergic reactions. Physicians documented the nature, frequency, management, and resolution of all AEs, performing causality assessments as needed.

Additional safety measures included comprehensive physical examinations, 12-lead electrocardiograms, neck ultrasounds, and pain assessments using the Visual Analog Scale. Submental skin laxity was evaluated using the SMSLG scale to track potential changes in skin condition.

### Sample Size

Based on the previous research,^13,14^ we assumed a 10.4% difference in the proportion of patients achieving a concurrent ≥2-grade improvement in CR-SMFRS and PR-SMFRS scores between the treatment and control groups, with an estimated placebo efficacy of 3%. Using an adaptive design with a 1-sided significance level of .025 and a power of 0.8, the patient allocation ratio between the treatment and placebo groups was set at 2:1. Sample size estimation was conducted using SAS (version 9.4; SAS Institute Inc.), resulting in a required total of 261 patients, with 174 in the treatment group and 87 in the placebo group. Accounting for a 20% dropout rate, the final sample size required is 324 patients, with 216 in the treatment group and 108 in the placebo group.

### Statistical Analysis

For the assessment of the primary endpoints, we used the *Z*-test and computed the absolute risk difference with 95% CI between the 2 groups using the Newcombe method.^15^ Superiority was concluded if the lower limit of the 95% CI was >0. A Cochran–Mantel–Haenszel analysis stratified by site and baseline value was performed for responder endpoints, whereas the analysis of covariance test was used to assess the continuous outcome variables. The analysis of continuous variables utilized paired *t*-tests or Wilcoxon signed-rank tests, whereas categorical variables were assessed using either χ^2^ tests or Fisher's exact tests. The statistical tests were conducted at a significance level of 5% on both sides. All statistical analyses were conducted using SAS (version 9.4; SAS Institute Inc.).

## RESULTS

### Demographic and Baseline Characteristics of the Patients

**Figure 2.**
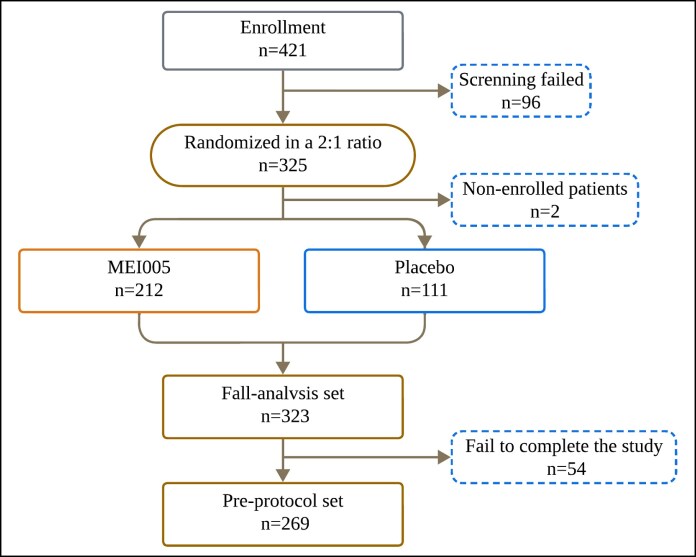
Flowcharts depicting the patients included in this study.

This study randomized 325 patients, of whom 323 received the study medication and were included in the full analysis set (FAS; Figure 2). Two patients did not receive treatment postrandomization and were excluded from the analysis. The mean age of participants was 34.55 ± 8.98 years (range, 18-62 years). The study population consisted of 70 males (21.7%) and 253 females (78.3%). Among these, 212 patients received MEI005 injections, and 111 patients received placebo treatment. Of the 325 enrolled patients, 281 completed the trial and 44 withdrew prematurely. The mean follow-up time was 192.98 ± 64.95 days, and the median was 181.00 days, with an interquartile range from 166.00 to 228.00 days.

In the FAS, the mean ages of patients receiving MEI005 injections and those in the placebo group were 34.06 ± 8.63 and 35.50 ± 9.58 years, respectively. Their mean heights were 164.64 ± 7.70 and 163.87 ± 7.52 cm, and their mean weights were 68.03 ± 13.91 and 66.07 ± 12.38 kg, respectively. The majority of participants in both groups were female, with 95.8% and 93.7% being of Han ethnicity in the deoxycholic acid injection and placebo groups, respectively. At baseline, patients in each trial group were evenly distributed in terms of CR-SMFRS, PR-SMFRS, PR-SMFIS, SMF volume (measured by MRI or vernier caliper), and SSRS (Table 2).

**Table 2.** Demographic and Baseline Characteristics of the Patients

Variable	Placebo (*n* = 111)	MEI005 (*n* = 212)	*P*-value
Age (SD), years	35.50 ± 9.58	34.06 ± 8.63	.172
Female, *n* (%)	86 (77.5)	167 (78.8)	.788
Han nationality, *n* (%)	104 (93.7)	203 (95.8)	.418
Height (SD), cm	163.87 ± 7.52	164.64 ± 7.70	.385
Weight (SD), kg	66.07 ± 12.38	68.03 ± 13.91	.214
BMI, mean (SD), kg/m^2^	24.48 ± 3.43	24.93 ± 3.66	.281
CR-SMFRS, *n* (%)			.827
Garde 2	73 (65.8)	142 (67.0)	
Garde 3	38 (34.2)	70 (33.0)	
PR-SMFRS, *n* (%)			.762
Garde 1	4 (3.6)	12 (5.7)	
Garde 2	67 (60.4)	127 (59.9)	
Garde 3	38 (34.2)	63 (29.7)	
Garde 4	2 (1.8)	10 (4.7)	
SMF by MRI (SD), mm^3^	5.14 ± 1.29	5.48 ± 1.54	.134
PR-SMFIS (SD)	42.41 ± 11.07	43.25 ± 10.15	.494
SMF by caliper (SD), mm	17.97 ± 6.76	19.38 ± 7.99	.113
SSRS, *n* (%)			.929
1	36 (32.4)	64 (30.2)	
2	35 (31.5)	64 (30.2)	
3	19 (17.1)	47 (22.2)	

BMI, body mass index; CR-SMFRS, Clinician-Reported Submental Fat Rating Scale; PR-SMFIS, Patient-Reported Submental Fat Impact Scale; PR-SMFRS, Patient-Reported Submental Fat Rating Scale; SD, standard deviation; SMF, submental fat; SSRS, Subject Self-Rating Scale.

### Treatment Characteristics

Patients in the MEI005 injection group received an average of 3.76 ± 1.31 treatments, whereas those in the placebo group received an average of 3.92 ± 0.89 treatments. There were no significant differences between the groups.

### Efficacy Evaluation

At 12 weeks following the final treatment, 18.9% (40/212) of patients in the MEI005 group showed simultaneous improvements of at least 2 grades on both the CR-SMFRS and PR-SMFRS scores, a significantly higher rate than the placebo group (1.8%; 2/111). Additionally, 38.7% (82/212) of patients in the MEI005 group achieved a ≥2-grade improvement on the CR-SMFRS, compared with 3.6% (4/111) in the placebo group. For the PR-SMFRS, 33.0% (70/212) of the MEI005 group achieved a ≥2-grade improvement, whereas only 5.4% (6/111) did so in the placebo group. All of these differences were statistically significant (*P* < .001), and sensitivity analyses confirmed the robustness of these results (*P* < .001).

**Figure 3.**
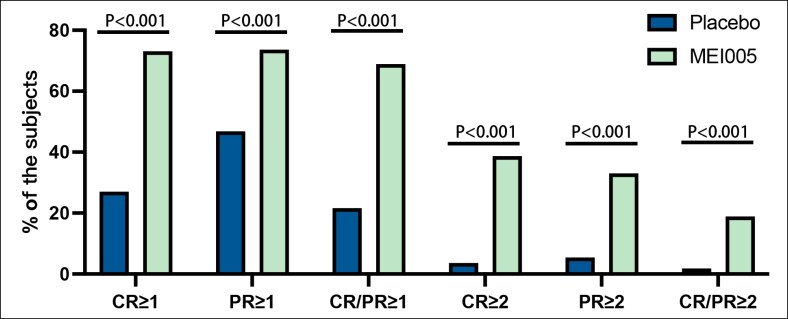
Percentages of patients who attained at least a 1- or 2-grade improvement on the Clinician-Reported Submental Fat Rating Scale or the Patient-Reported Submental Fat Rating Scale. CR, Clinician-Reported Submental Fat Rating Scale; PR, Patient-Reported Submental Fat Rating Scale; CR/PR, both CR and PR.

Furthermore, in the MEI005 group, 68.9% (146/212) of patients showed simultaneous improvements of at least 1 grade on both CR-SMFRS and PR-SMFRS scores, compared with 21.6% (24/111) in the placebo group. For the CR-SMFRS, 73.1% (155/212) of patients in the MEI005 group achieved a ≥1-grade improvement, whereas only 27.0% (30/111) in the placebo group. Similarly, for the PR-SMFRS, 73.6% (156/212) in the MEI005 group achieved a ≥1-grade improvement, compared with 46.8% (52/111) in the placebo group. Across all measures, the MEI005 group consistently showed superior improvement rates compared with the placebo group (*P* < .001; Figure 3, Table 3).

**Table 3.** Secondary and Additional Efficacy Endpoints Evaluated at 12 Weeks After the Last Treatment

Variables	Placebo (*n* = 66)	MEI005 (*n* = 120)
MRI responder^a^, *n* (%)	10 (15.2)	60 (50.0)

	Placebo (*n* = 103)	MEI005 (*n* = 179)
SMF reduction percentage by caliper, ±SD (%)	6.32 ± 1.71	21.42 ± 1.95
Reduction in PR-SMFIS scores, ±SD	4.68 ± 13.47	22.58 ± 17.47

	Placebo (*n* = 111)	MEI005 (*n* = 212)
CR-SMFRS ≥1-grade improvement, *n* (%)	30 (27.0)	155 (73.1)
PR-SMFRS ≥1-grade improvement, *n* (%)	52 (46.8)	156 (73.6)
CR-SMFRS ≥2-grade improvement, *n* (%)	4 (3.6)	82 (38.7)
PR-SMFRS ≥2-grade improvement, *n* (%)	6 (5.4)	70 (33.0)
SSRS responder, *n* (%)	17 (15.3)	125 (59.0)
SGQ responder, *n* (%)		
1 (fat under chin)	10 (9.0)	127 (59.9)
2 (definition between chin and neck)	11 (9.9)	109 (51.4)
3 (satisfaction with the treatment)	26 (23.4)	143 (67.5)

CR-SMFRS, Clinician-Reported Submental Fat Rating Scale; PR-SMFIS, Patient-Reported Submental Fat Impact Scale; PR-SMFRS, Patient-Reported Submental Fat Rating Scale; SD, standard deviation; SMF, submental fat; SSRS, Subject Self-Rating Scale; SGQ, Subject Global Questions.

^a^The reduction in SMF volume is at least 10% compared with the baseline.

**Figure 4.**
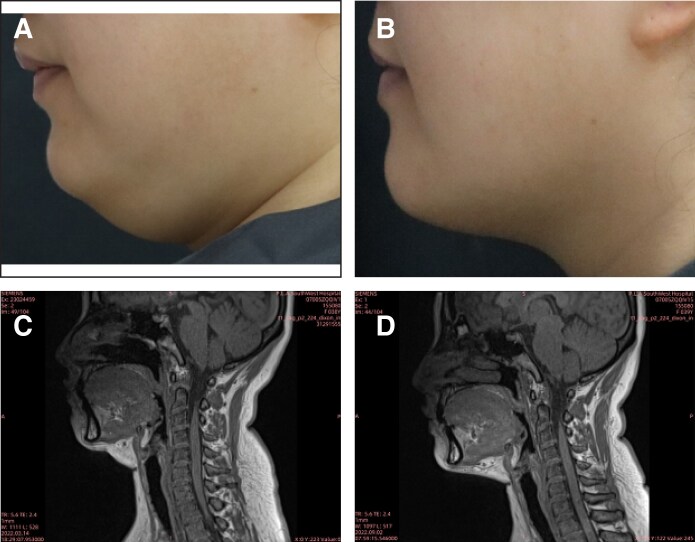
A 38-year-old female treated with MEI005. (A) and (B) reveal pretreatment and improved jawline contour, respectively, at 12 weeks after the fourth treatment session (Week 12), whereas (C) and (D) reveal reduced submental fat volume on MRI at the same time points.

MRI examinations were completed by 186 patients (placebo: *c*66; MEI005: *n* = 120). At 12 weeks, 50.0% (60/120) of patients in the MEI005 group showed at least a 10% reduction in SMF volume from baseline, significantly higher than the 15.2% (10/66) in the placebo group (*P* < .001; Table 3). Figure 4 presents a typical case of a patient treated with MEI005, showing improved jawline contour corroborated by reductions in SMF volume on MRI.

PR-SMFIS scores were collected from 282 patients (placebo: *n* = 103; MEI005: *n* = 179). The total PR-SMFIS score reduction from baseline was 22.58 ± 17.47 in the MEI005 group, significantly greater than the reduction observed in the placebo group (4.68 ± 13.47; *P* < .001; Table 3).

Vernier caliper measurements were also completed by 282 patients (placebo: *n* = 103; MEI005: *n* = 179). The mean percentage reduction in SMF thickness from baseline was 21.42 ± 1.95% in the MEI005 group, compared with 6.32 ± 1.71% in the placebo group (*P* < .001).

**Figure 5.**
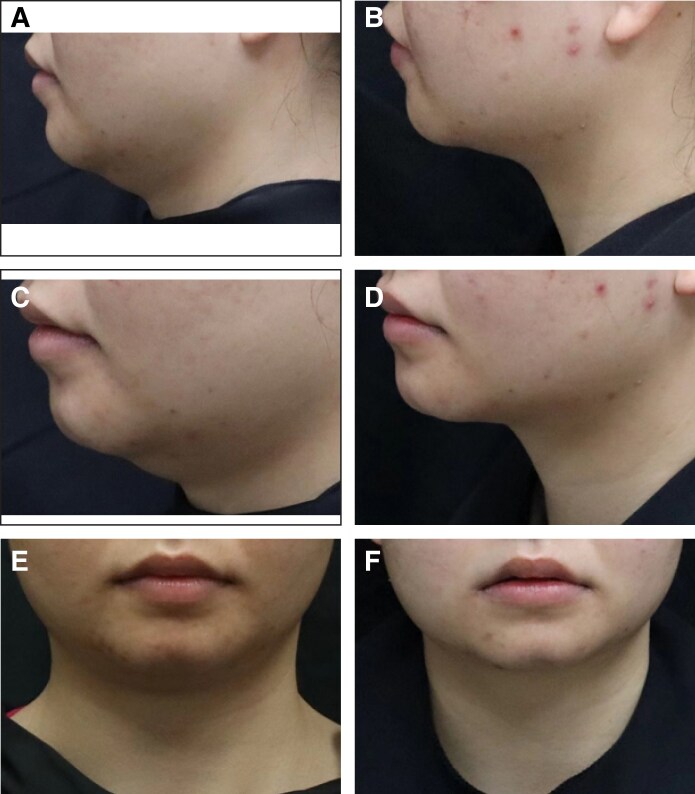
Representative case receiving MEI005 injection. (A, C, E) Lateral, frontal, and oblique views of the jawline contour of a 26-year-old female at baseline, and (B, D, F) the corresponding views 12 weeks after the third treatment session.

**Figure 6.**
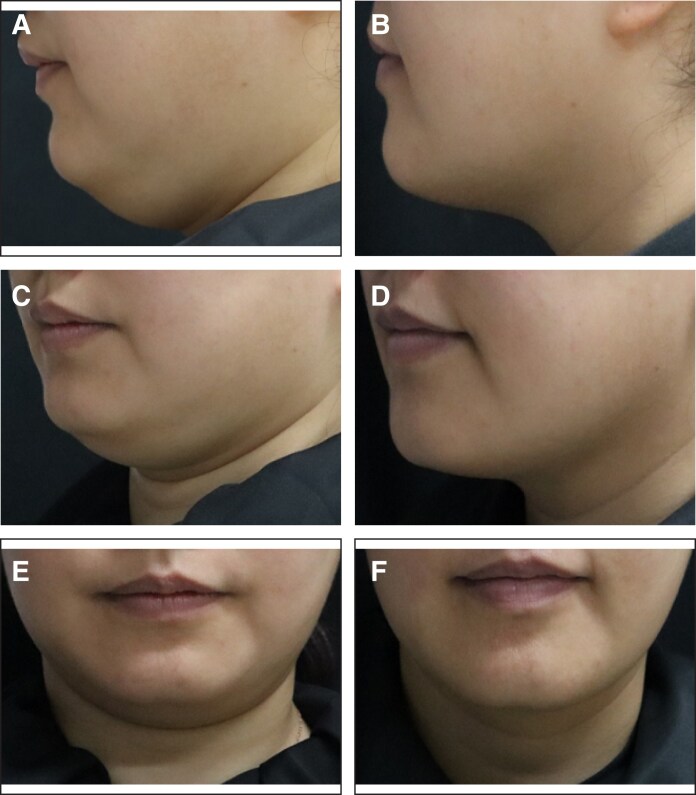
Representative case receiving MEI005 injection. (A, C, E) Lateral, frontal, and oblique views of the jawline contour of a 38-year-old female at baseline, and (B, D, F) the corresponding views 12 weeks after the fourth treatment session.

**Figure 7.**
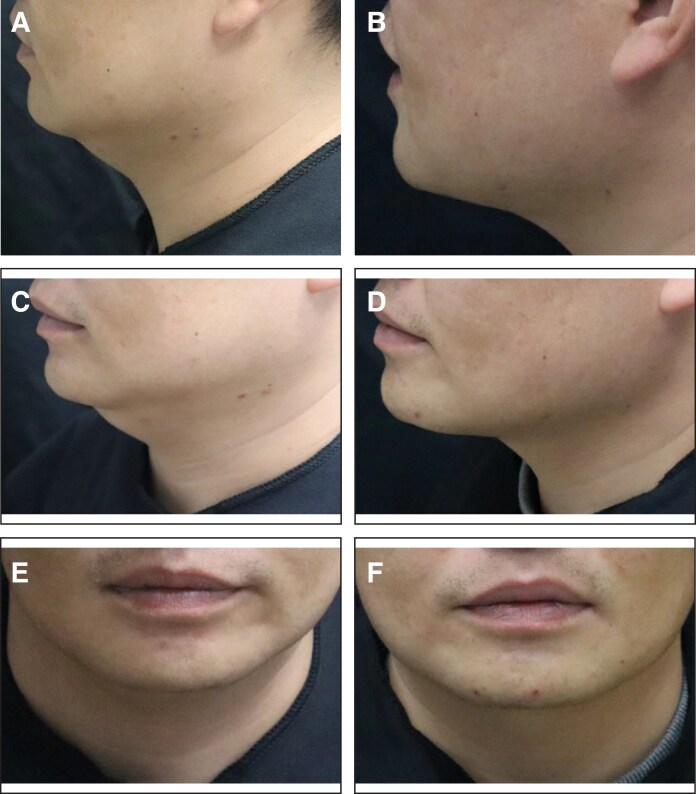
Representative case receiving MEI005 injection. (A, C, E) Lateral, frontal, and oblique views of the jawline contour of a 34-year-old male at baseline, and (B, D, F) the corresponding views 12 weeks after the third treatment session.

**Figure 8.**
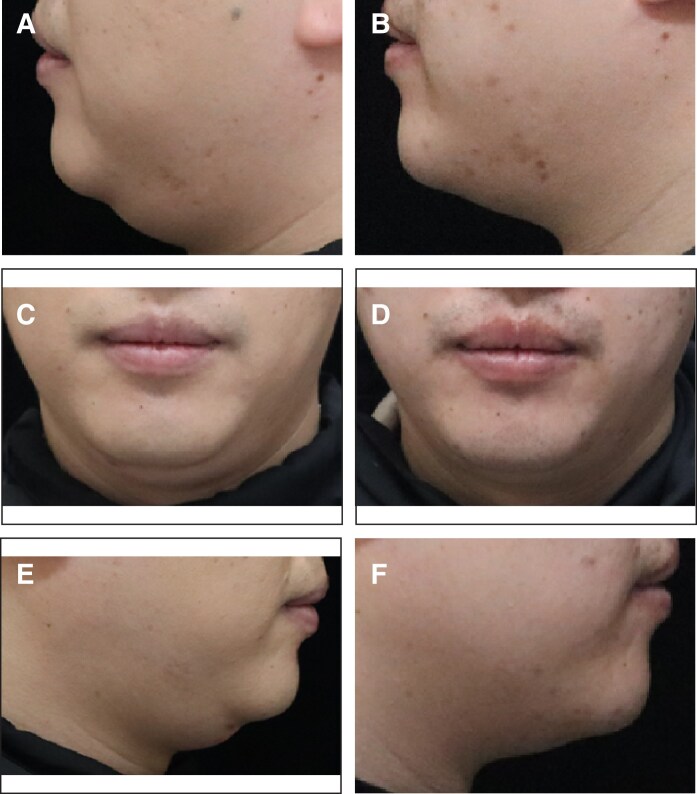
Representative case receiving MEI005 injection. (A, C, E) Lateral and frontal views of the jawline contour of a 32-year-old male at baseline, and (B, D, F) the corresponding views 12 weeks after the fourth treatment session.

Additionally, the proportion of patients with an SSRS score of ≥4 was 15.3% (17/103) in the placebo group and 59.0% (125/212) in the MEI005 group, indicating significantly higher satisfaction in the MEI005 group (*P* < .001). The MEI005 group also had a significantly higher effective response rate on the SGQ (SGQ1: 127/212, 59.9%; SGQ2: 109/212, 51.4%; SGQ3: 143/212, 67.5%) compared with the placebo group (SGQ1: 10/111, 9.0%; SGQ2: 11/111, 9.9%; SGQ3: 26/111, 23.4%; *P* < .001). Secondary and additional efficacy endpoints assessed at 12 weeks postfinal treatment are summarized in Table 3. Figures 5-8 present pretreatment and 12 weeks posttreatment photographs from 4 patients, providing a visual representation of the treatment outcomes.

### Safety Evaluation

The first follow-up visit was conducted on-site at the hospital 1 week after the initial treatment, whereas follow-ups 1 week after each subsequent treatment were conducted through telephone. The number of AEs occurrences in the MEI005 were 211 cases (99.5%) and 108 cases (97.3%) in the placebo group. There is no significant difference (*P* = .233). Approximately 95.7% of AEs in the MEI005 group and 96.5% of AEs in the placebo group showed improvement or complete resolution within 15 days. By the end of the study, ∼96.2% of AEs in the MEI005 group and 98.2% in the placebo group had improved or fully resolved.

The severity of AEs and injection-related AEs whose incidence rate were over 10% were all listed in Table 4. The majority of AEs in both groups were mild and moderate, with 97.3% and 26.1% in the placebo group and 99.1% and 37.3% in the MEI005 group. Among injection-related AEs in the MEI005 group, the most common side effect was pain (211/212, 99.5%), followed by swelling (168/212, 79.2%), numbness (73/212, 34.4%), bruising (60/212, 28.3%), induration (60/212, 28.3%), and edema (22/212, 10.4%). In the placebo group, injection-related AEs mainly included pain (98/111, 88.3%), bruising (29/111, 26.1%), and swelling (23/111, 20.7%).

**Table 4.** Severity of AEs and AEs With Incidence Rate Over 10%

AE, *n* (%)	Placebo, *n* = 111	MEI005, *n* = 212
Severity^a^		
Mild	108 (97.3)	210 (99.1)
Moderate	29 (26.1)	79 (37.3)
Severe	4 (3.6)	3 (1.4)
Injection-related AEs (>10%), *n* (%)		
Pain	98 (88.3)	211 (99.5)
Hematoma (bruising)	29 (26.1)	60 (28.3)
Swelling	23 (20.7)	168 (79.2)
Anesthesia (numbness)	NA	73 (34.4)
Induration	NA	60 (28.3)
Edema	NA	22 (10.4)

AE, Adverse event; NA, not available.

^a^Mild: asymptomatic or mild symptoms; clinical or diagnostic observations only; intervention not indicated. Moderate: minimal, local, or noninvasive intervention indicated; limiting age-appropriate instrumental activities of daily living. Severe: medically significant but not immediately life-threatening; hospitalization or prolongation of hospitalization indicated; disabling; limiting self-care activities of daily living.

In this trial, a total of 9 serious AEs unrelated to the investigational medication occurred. Among these, 4 cases were reported in the MEI005 group (including open wounds on the hand, thyroid cancer, acute urticaria, and a nasopharyngeal mass), whereas 5 cases were observed in the placebo group (including gallstones, vocal cord cyst, trigeminal neuralgia, sinus tachycardia, and an intrathecal mass). In addition, skin laxity was unchanged or improved in 90.1% of MEI005 group and 92.8% of placebo group.

## DISCUSSION

The accumulation of excess SMF is a common concern that leads many patients to seek aesthetic treatments. As SMF increases, individuals may be perceived less favorably, appearing less likable, intelligent, and agreeable.^16^ Compared with complex surgical procedures, patients show a strong preference for nonsurgical fat reduction methods, such as lipolytic drug injections, because of reduced pain, minimal scarring, and shorter recovery times.^17^ Deoxycholic acid, a cell-disrupting agent, physically disrupts cell membranes when injected into adipose tissue, leading to the dissolution of fat cells.^18,19^ Studies have shown that this localized dissolution attracts macrophages to clear cellular debris and lipids, followed by neutrophilic infiltration, cell-mediated fibroblast activation, and collagen deposition, thereby strengthening the structural integrity of the underlying tissue.^20-22^ After the inflammation resolves, fibrotic septal thickening, neovascularization, and fat lobule atrophy remain.^23^

In this randomized, double-blind, placebo-controlled, multicenter clinical trial, we evaluated the efficacy and safety of MEI005, with deoxycholic acid as the active ingredient, in patients with moderate-to-severe SMF accumulation. The primary efficacy endpoint evaluates the combined improvement in CR-SMFRS and PR-SMFRS scores after 12 weeks of the last treatment. A significantly higher proportion of patients in the MEI005 group demonstrated improvements compared with the placebo group. Additionally, ATX101 (Belkyra/Kybella), approved by the US FDA and EMA for SMF reduction, has been evaluated in 4 Phase III clinical trials.^11-14^ Compared with placebo, ATX-101 showed a higher proportion of patients achieving at least a 1-point improvement in CR-SMFRS scores (58.3% and 62.3% vs 34.5%, and 59.2% and 65.3% vs 23.0%).^11,12^ Similarly, a greater proportion of patients treated with ATX-101 showed ≥1-point improvement in both CR-SMFRS and PR-SMFRS (70.0% vs 18.6% and 66.5% vs 22.2%),^13,14^ and the proportion achieving ≥2-point improvement was also higher (13.4% vs < 0.1% and 18.6% vs 3.0%).^11-14^ Most AEs were mild, transient, resolved without sequelae, and were associated with the treatment area.^11-14^ Comparing the results of these trials, the improvement in SMF with MEI005 also appears significant and consistent, demonstrating its efficacy and safety as a nonsurgical treatment option.

MRI and vernier caliper measurements offer objective indicators of SMF volume reduction.^24^ Following 12 weeks of the last treatment, a notably greater proportion of patients in the MEI005 group demonstrated at least 10% reductions in submental volume compared with baseline, as verified by MRI and vernier calipers. This suggests that MEI005 not only impacts outward appearance but also leads to a reduction in fat volume.

The visual and emotional impact of SMF was assessed using the PR-SMFIS and the SSRS scores. The reduction in PR-SMFIS score and the effective response rate of SSRS were significantly higher in the MEI005 group compared with the placebo group, indicating greater psychological improvement and satisfaction.

In terms of safety, common AEs associated with the injection included mild-to-moderate pain, swelling, and bruising. These local reactions are associated with the injection route, the pharmacological action of deoxycholic acid, and the tissue responses it induces.^25-27^ Injection technique and management strategies also play a role in the occurrence of these events.^28,29^ Although the MEI005 group experienced a higher incidence of AEs than the placebo group, most events were mild to moderate and resolved without intervention in a short period, indicating a generally favorable safety profile. To further manage and reduce discomfort, several measures can be taken. Applying cold packs before and after treatment, using topical and injectable lidocaine, taking antihistamines and anti-inflammatory medication, and wearing a chin strap for support to alleviate pain and discomfort.^23^ Additionally, careful injection technique and pretreatment and immediate posttreatment cooling are recommended to reduce swelling.^23,28^

The rapid growth of China's aesthetic medicine market has driven an increasing demand for reliable products.^30^ This study contributes to the growing body of evidence supporting the efficacy and safety of injectable deoxycholic acid for SMF reduction, addressing a significant gap in the Chinese population. Although previous trials, such as the initial ATX-101 studies conducted in North American and European populations, have demonstrated efficacy,^11-14^ this represents the first rigorous clinical evaluation of a related product in China. With the rising preference for nonsurgical alternatives to liposuction in SMF reduction, this large, multicenter, randomized controlled trial provides robust data to inform clinical practice and potentially guide regulatory decisions for similar products in the region.

This study has several limitations that should be acknowledged. First, the follow-up period was relatively short, lasting <6 months, which precludes assessment of the long-term durability of treatment efficacy and the potential occurrence of delayed AEs. Second, the unequal sample sizes between the treatment and placebo groups may have an impact on the precision of the results, but this is unlikely to affect the overall reliability of the study. Finally, the study population was limited to Chinese adults, which may restrict the generalizability of the findings to other ethnic groups with differing anatomical, metabolic, or aesthetic preferences.

## CONCLUSIONS

The results of this Phase III study demonstrated that MEI005 is effective in improving moderate-to-severe SMF accumulation in Chinese adults. The treatment showed a high satisfaction and safety profile based on clinician assessments, patient reports, and objective measurements. For patients seeking a minimally invasive procedure, MEI005 offers a relatively simple injectable approach to reducing unwanted SMF.

## Supplemental Material

This article contains supplemental material located online at https://doi.org/10.1093/asj/sjaf031.

## Supplementary Material

sjaf031_Supplementary_Data
