# Supplementary figures and images for: A Randomized, Double-Blind, Placebo-Controlled, Multicentered Study to Evaluate the Efficacy and Safety of MEI005 in Reducing Submental Fat in Chinese Adults
Source: Aesthet Surg J. 2025 Mar 4;45(6):629–37. doi: 10.1093/asj/sjaf031 (PMC12080881; doi:10.1093/asj/sjaf031)

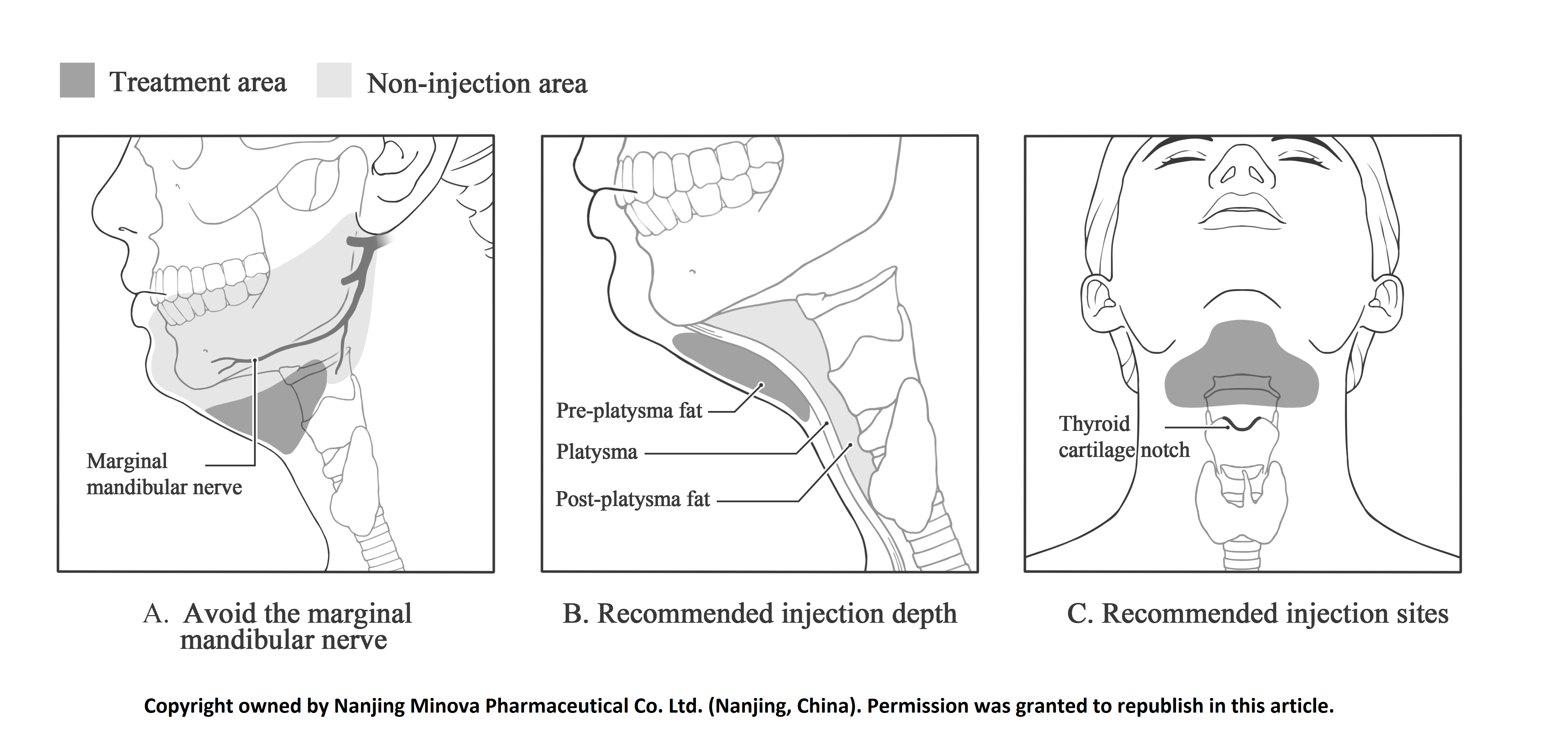

Supplement: sjaf031_Supplementary_Data [file sjaf031_supplementary_data.zip › Figure_S1.jpg]

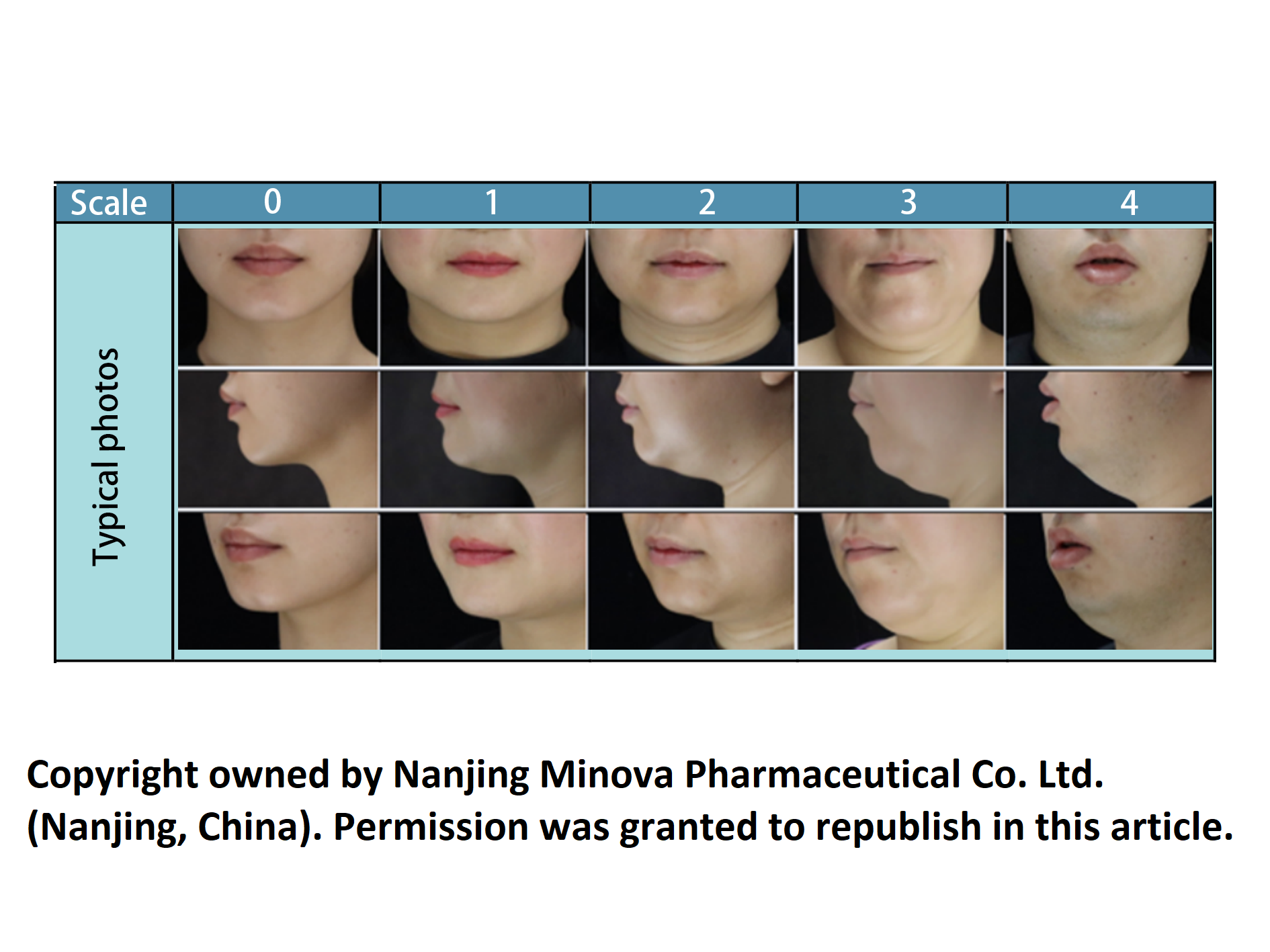

Supplement: sjaf031_Supplementary_Data [file sjaf031_supplementary_data.zip › Figure_S2.png]

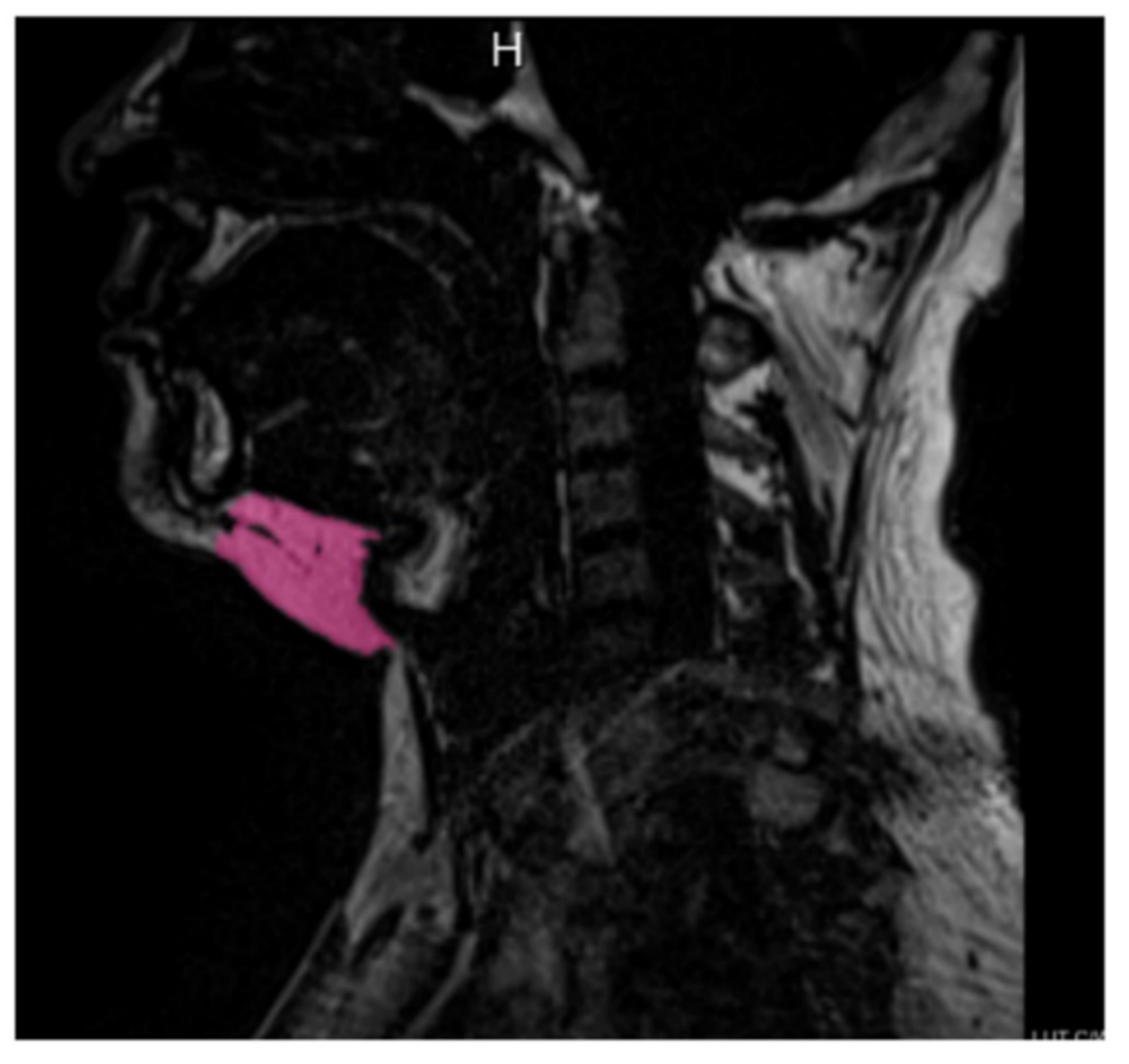

Supplement: sjaf031_Supplementary_Data [file sjaf031_supplementary_data.zip › Figure_S3A (2).png]
